# A Vaccinology Approach to the Identification and Characterization of *Dermanyssus gallinae* Candidate Protective Antigens for the Control of Poultry Red Mite Infestations

**DOI:** 10.3390/vaccines7040190

**Published:** 2019-11-20

**Authors:** José Francisco Lima-Barbero, Marinela Contreras, Lourdes Mateos-Hernández, Francisco Manuel Mata-Lorenzo, Roxana Triguero-Ocaña, Olivier Sparagano, Robert D. Finn, Christina Strube, Daniel R.G. Price, Francesca Nunn, Kathryn Bartley, Ursula Höfle, Mariana Boadella, Alasdair J. Nisbet, José de la Fuente, Margarita Villar

**Affiliations:** 1 SaBio. Instituto de Investigación en Recursos Cinegéticos, IREC (CSIC-UCLM-JCCM), Ronda de Toledo 12, 13071 Ciudad Real, Spain; JoseFco.Lima@alu.uclm.es (J.F.L.-B.); Marinela.Contreras@uclm.es (M.C.); fcomanuelmata@gmail.com (F.M.M.-L.); Roxana.Triguero@uclm.es (R.T.-O.); ursula.hofle@uclm.es (U.H.); 2 Sabiotec, S.A. Ed., Polivalente UCLM, Camino de Moledores, 13005 Ciudad Real, Spain; mariana@sabiotec.es; 3 UMR BIPAR, INRA, Ecole Nationale Vétérinaire d´Alfort, ANSES, Université Paris-Est, 94700 Maisons-Alfort, France; lourdes.mateos@vet-alfort.fr; 4 Department of Infectious Diseases and Public Health, City University of Hong Kong, Kowloon, Hong Kong SAR, China; Olivier.sparagano@cityu.edu.hk; 5 Department of Applied Sciences, Faculty of Health & Life Sciences, Northumbria University, Newcastle Upon Tyne NE1 8ST, UK; RFinn@sgu.edu; 6 St George’s International School of Medicine, Keith B. Taylor Global Scholars Program, Northumbria University, Newcastle NE1 8ST, UK; 7 Institute for Parasitology, Centre for Infection Medicine, University of Veterinary Medicine Hannover, 30559 Hannover, Germany; Christina.Strube@tiho-hannover.de; 8 Moredun Research Institute, Pentlands Science Park, Bush Loan, Edinburgh, Midlothian EH26 0PZ, UKfrancesca.nunn@moredun.ac.uk (F.N.); kathryn.bartley@moredun.ac.uk (K.B.); alasdair.nisbet@moredun.ac.uk (A.J.N.); 9 Department of Veterinary Pathobiology, Center for Veterinary Health Sciences, Oklahoma State University, Stillwater, OK 74078-2007 USA; 10 Biochemistry Section, Faculty of Science and Chemical Technologies, and Regional Centre for Biomedical Research [CRIB], University of Castilla-La Mancha, 13071 Ciudad Real, Spain

**Keywords:** *Dermanyssus*, poultry red mite, proteomics, vaccine, vaccinology, control, protective antigens

## Abstract

The poultry red mite (PRM), *Dermanyssus gallinae*, is a hematophagous ectoparasite considered as the major pest in the egg-laying industry. Its pesticide-based control is only partially successful and requires the development of new control interventions such as vaccines. In this study, we follow a vaccinology approach to identify PRM candidate protective antigens. Based on proteomic data from fed and unfed nymph and adult mites, we selected a novel PRM protein, calumenin (Deg-CALU), which is tested as a vaccine candidate on an on-hen trial. *Rhipicephalus microplus* Subolesin (Rhm-SUB) was chosen as a positive control. Deg-CALU and Rhm-SUB reduced the mite oviposition by 35 and 44%, respectively. These results support Deg-CALU and Rhm-SUB as candidate protective antigens for the PRM control.

## 1. Introduction

The parasitic mite *Dermanyssus gallinae* (Mesostigmata: Dermanyssidae), also known as poultry red mite (PRM), is the major pest for the poultry industry [1]. It is distributed worldwide, present in every production system and can be shown in high prevalence in commercial egg laying facilities, as is the case in Europe, reaching a prevalence of 100% in some countries [2]. The mite feeds on the blood of the hens in the dark and spends just 30–60 min on the host [3]; during daytime it hides in cracks out of the reach of the birds [4]. According to this behaviour, PRM can be better considered as a micro predator [5]. *Dermanyssus gallinae* requires a blood meal for moulting from protonymph to deutonymph, to adult and for egg-laying [6]. The PRM has severe effects on hens´ health and welfare as its presence is related to anaemia and stress. Depending on the level of infestation, it can lead to increased mortality by exsanguination and behavioural disorders due to sleep deprivation [7,8,9]. Besides, PRM can act as a vector for several pathogenic viruses and bacteria [10,11]. The threat for the industry relies on the economic losses caused by the PRM in two ways: reduction of egg production associated with a negative impact on feed conversion ratio and a higher proportion of blood-spotted and low-quality eggs, which are downgraded and, secondly, cost-derived from pest control actions [12]. A recent estimate has set the losses caused by the PRM, only in the European Union, at €231 million [13].

One of the biggest challenges facing the egg industry is the control of the PRM. Chemical treatments have traditionally been used to control PRM, however, there is a limited number of acaricides authorised to treat mite infestations by European or national legislation. In addition, the development of resistance reduces the efficiency of the commonly used acaricides [2,14]. Recent research is focused on exploring different tools for alternative control of poultry red mite as biological control, plant-derived products, entomopathogenic fungi or physical control [2].

Vaccine development is a novel, environmentally friendly and promising method for PRM control. Some advantages of vaccination are the reduction of the use of pesticides, no contamination of the environment and animal products and unlikely development of resistance by the parasites [15]. Vaccination implies the identification of proteins that can act as vaccine antigens [16]. Recombinant proteins have been used to immunise host animals against parasitic species. A recombinant form of Bm86 is part of commercial vaccines that can protect cattle from *Boophilus microplus* tick infestations [17]. The limits to vaccine development are partly because of limited information about constituent proteins of *Dermanyssus gallinae* [18,19]. The publication of the transcriptome [20] and, more recently, the PRM genome [21] is expected to enhance protein identification with immunisation purposes. Proteins such as the tick Subolesin, the ortholog of the akirin present in insects and vertebrates, have shown a reducing effect on infestations by several ectoparasites, PRM amongst them [15,22].

In this work, we analyzed for the first time the proteomes of fed and unfed *D. gallinae* adults and nymphs. Quantitative proteomics analysis led to the identification of differentially represented proteins between different development stages and feeding status that could be putative protective antigens against *D. gallinae.* The efficacy of a candidate antigen was tested through an experimental infestation of vaccinated and naïve hens. 

## 2. Materials and Methods 

### 2.1. Mite Collection and Proteins Extraction

*D. gallinae* were collected, and proteins were extracted as previously described [23]. Briefly, mites collected from a free-range poultry unit in North Eastern England were distributed in four groups: engorged female adult mites (FA), non-fed female adult mites (UA), engorged proto- and deutonymphs (FN), and unfed proto- and deutonymphs (UN). For protein extraction, each group of mites were resuspended in ice-cold PBS supplemented with cOmplete Protease Inhibitor Cocktail (Roche Diagnostics GmbH, Mannheim, Germany) and homogenised on ice for two pulses of 30 s each with Ultra Turrex^®^ T 25 D-S2 with a S25N-8G dispersing element (IKA, Sataufen, Germany). Samples were centrifuged at 5000× *g* for 20 min at 4 °C to remove insoluble material and debris. Protein concentration in the soluble phase was determined using the BCA Protein Assay (Thermo Scientific, San Jose, CA, USA) with bovine serum albumin as standard and protein samples were snap-frozen and stored at −80 °C until proteomics analysis.

### 2.2. Proteomics Data Acquisition and Analysis

Protein extracts from the four groups of mites (75 µg per sample) were in-gel concentrated, as previously described [24]. After visualization of the unseparated protein bands by GelCode Blue Stain Reagent (Thermo Scientific), bands were excised and digested overnight at 37 °C with 60 ng/μL sequencing grade trypsin (Promega, Madison, WI, USA) at 5:1 protein:trypsin (w/w) ratio in 50 mM ammonium bicarbonate pH 8.8, containing 10% acetonitrile (v/v) [25]. The resulting tryptic peptides were extracted with 12 mM ammonium bicarbonate pH 8.8, and digestion was stopped adding trifluoroacetic acid to a final concentration of 1%. Peptides were desalted onto OMIX Pipette tips C18 (Agilent Technologies, Santa Clara, CA, USA), dried-down and stored at −20 °C until mass spectrometry analysis.

The desalted protein digests were resuspended in 0.1% formic acid and analyzed by reverse-phase liquid chromatography coupled to mass spectrometry (RP-LC-MS/MS) using an Easy-nLC II system coupled to an LTQ-Orbitrap-Velos-Pro mass spectrometer (Thermo Scientific) as previously described [24]. Briefly, after on-line concentration of peptides by RP using a 0.1 × 20 mm C18 RP precolumn (Thermo Scientific), peptides were separated in a 0.075 × 250 mm C18 RP column (Thermo Scientific) operating at 300 nL/min and eluted using a 140-min gradient from 5 to 40% solvent B in solvent A (solvent A: 0.1% formic acid in water; solvent B: 0.1% formic acid in acetonitrile). ESI ionisation was done using a Nano-bore Stainless Steel emitter ID 30 µm (Thermo Scientific) interface. Peptides were detected in survey scans from 400 to 1600 amu (1 µscan), followed by 20 data-dependent MS/MS scans (Top 20), using an isolation width of 2 mass-to-charge ratio units, normalised collision energy of 35%, and dynamic exclusion applied during 30 s periods. 

The MS/MS raw files were searched against a compiled database containing the UniProt Parasitiformes and *Gallus gallus* proteome databases (141,928 and 29,484 entries in April 2018, respectively) (http://www.uniprot.org) together with a database created from the predicted secretome and transmembrane proteins of *D. gallinae* (10,454 entries) [26], using the SEQUEST algorithm (Proteome Discoverer 1.4, Thermo Scientific). The constraints imposed for the search were: tryptic cleavage after Arg and Lys, two maximum missed cleavages, tolerances of 20 ppm and 0.05 Da for precursor and MS/MS fragment ions, respectively, and Met oxidation and Cys carbamidomethylation as variable modifications. Searches were also performed against a decoy database in an integrated decoy approach. A false discovery rate (FDR) < 0.01 was considered as a condition for successful peptide assignments and at least two peptide-spectrum matches (PSMs) per protein in at least one of the samples analyzed were the necessary condition for protein identification. Three biological replicates were used for each of the four groups of PRM analyzed (fed and unfed adults and nymphs). Raw proteomics data are available through the PeptideAtlas repository (http://www.peptideatlas.org) with the dataset identifier PASS01346.

Gene ontology (GO) analysis of biological process (BP), molecular function (MF) and cellular component (CC) was conducted using Blast2GO software (version 3.0; www.blast2go.com) and manually completed using QuickGO databases (https://www.ebi.ac.uk/QuickGO/). To show proteins distribution amongst the four groups, a Venn´s diagram was constructed using InteractiveVenn (http://www.interactivenn.net/) [27].

### 2.3. Criteria for Selection of Candidate Protective Antigens

The selection of candidate protective antigens to be tested was based on differentially over-represented proteins in adult female *versus* nymph mites of the same feeding status and/or differentially over-represented proteins in fed *versus* unfed mites in the same developmental stage, since these proteins are potentially good candidates to be used as antigens to block mites development. For quantitative analysis of PRM proteins, after discarding host proteins, the total number of PSMs for each PRM protein was normalised against the total number of PSMs in PRM samples and compared between groups using a paired comparison Chi^2^-test (**χ^2^**) (*p* < 0.05) in R software [28] (Table 1). After discarding differentially represented proteins that had been tested before as vaccine candidates: vitellogenin and cathepsin [18], tropomyosin and paramyosin [29] and histamine release factor [30]; proteins identified in a database from secreted and transmembrane *D. gallinae* proteins were selected to favour antibody-antigen interactions in mites feeding on vaccinated hens. Of the potential candidates, only calumenin fulfilled the criteria and was selected to be tested as a protective antigen. Calumenin isoform 2 coding sequence (isotig18930) was obtained from the published predicted secretome and transmembranome of *D. gallinae* [26]. As a vaccination positive control, Subolesin from *Rhipicephalus microplus* (Rhm-SUB) [22]. 

### 2.4. Cloning of Antigens and Production of Recombinant Proteins

Recombinant *Rhipicephalus microplus* Subolesin (Rhm-SUB), with GenBank accession number GQ456170 was expressed and purified as reported previously [31]. *D. gallinae* calumenin (Deg-CALU) (GenBank accession number BK011287) cDNA was amplified from a synthetic gene optimised for codon usage in *E. coli* (GenScript, Hong Kong) using oligonucleotide primers Deg-CALU-F: 5′- CACCATGGATAACTACGTCGATCAG and Deg-CALU-R: 5′- GAGTTCGCTGTGCTGGCCGA. The DNA coding sequence was cloned in the expression vector pET101, expressed in *E. coli* strain BL21 as previously described [32]. Transformed *E. coli* strains were induced with isopropyl β-D-1-thiogalactopyranoside (IPTG) for 4.5 h to produce recombinant proteins, which using this expression system were fused to Histidine tags for purification by affinity to Ni using 1 mL HisTrap FF columns mounted on an AKTA-FPLC system (GE Healthcare, Piscataway, NJ, USA) in the presence of 7 M urea lysis buffer [32] and were purified to >95% of total cell.

### 2.5. Sequence Analysis for Deg-CALU

Calumenin coverage of identification by RP-LC-MS/MS was investigated. Analysis of protein sequence identity for Deg-CALU was conducted using the Blastp tool from BLAST (https://blast.ncbi.nlm.nih.gov/Blast.cgi).

### 2.6. Vaccine Formulations

The purified denatured recombinant proteins were refolded by dialysis against 1000 volumes of PBS (137 mM NaCl, 2.7 mM KCl, 10 mM Na_2_HPO_4_, 1.8 mM KH_2_PO_4_), pH 7.4 for 12 h at 4 °C. Recombinant proteins were then concentrated using an Amicon Ultra-15 ultrafiltration device (cut off 10 KDa) (Millipore-Merck, Darmstadt, Germany), adjusted to 0.5 mg/mL. For vaccine formulation, recombinant Rhm-SUB and Deg-CALU proteins or saline control were adjuvanted in Montanide ISA 71 VG (Seppic, Paris, France) [33,34].

### 2.7. Hen Vaccination and PRM Infestation

Three experimental groups (Rhm-SUB, Deg-CALU and Control) with 5 Lohmann Brown 20 weeks old hens each were randomly assigned. One hen in the control group was humanely euthanized on day 28 due to health problems not related to the experiment. Hens were vaccinated on day 0 (V1) and 14 (V2) with a 0.4 mL intramuscular injection in the breast muscle. Total vaccine doses were 20 µg and 40 µg, V1 and V2 respectively, for Deg-CALU and 45 µg for both vaccinations of Rhm-SUB. The study was terminated on day 42.

An on-hen feeding device [35] was used to evaluate vaccine efficiency. Briefly, the device consists of a pouch made from flexible 105 µm aperture width polyester mesh, containing approximately 100 starved adult female *D. gallinae* mites. Mites were starved for 1 week at room temperature and 3 weeks at 4 °C and were placed in the feeding devices and attached for 3 h to the defeathered thigh of each hen using a small amount of medical tape (Leucoplast, BSN Medical, Germany) and secured with a medical bandage (Henry Schein Inc., New York, EEUU). Feeding assays were conducted three times (replicates), at days 28, 30 and 32 post V1 [34]. Every hen was included in each replicate.

### 2.8. Vaccine Efficacy

Mites were identified as fed when they were engorged and bright red. They were considered dead if they did not show any movement and were unresponsive to touch stimulus. Engorged mites were collected from the pouches and transferred into individual wells of a 96-well tissue culture plate (Costar, Corning, NY, USA) and sealed with AeraSeal film (Sigma-Aldrich, St. Louis, USA; A9224-50EA). Plates were placed into an incubator (25 °C and 85% relative humidity) and checked every 24 h for five days and on day 7 after feeding. Feeding rate was recorded when transferring from the pouch to the plates. Mite mortality, laying mites, oviposition, egg hatchability and larval development were recorded in every check to assess the vaccine efficacy.

### 2.9. Analysis of Hen IgY Antibody Response by ELISA

Blood samples were collected from each hen before each vaccination (day 0 and 14), during feeding assays (day 28) and at the end of the experiment. Whole blood was obtained by venipuncture of the cutaneous ulnar vein. Serum was recovered following clotting at 4 °C for 24 h and centrifugation at 3000 g and stored at −20 °C for further analysis.

Two indirect ELISA tests were performed based on previously described protocols [36] to detect IgY antibodies against both antigens in serum samples from vaccinated and control hens. One was performed to evaluate the trend on the antibody level during the whole experiment and another to compare the level of antibodies and the mite oviposition at the moment of experimental infestation. Briefly, high absorption capacity polystyrene microtiter 96-well plates were coated overnight at 4 °C with 0.5 µg/well of each recombinant protein diluted to 10 µg/mL with coating buffer (50 mM sodium bicarbonate, pH 9.6). ELISA plates were washed six times with 200 µL PBST (PBS containing 0.05% v/v Tween-20) and blocked with 200 µL/well of blocking buffer (10% w/v) powdered soy milk in TBST buffer (50 mM Tris, 150 mM NaCl, 0.05% v/v Tween-20) for 2 h at room temperature on shaker. Sera from the vaccinated hens were used as primary antibodies. 50 µL/well of the primary antibodies were used at a 1/1600 (Rhm-SUB and pooled control group) or 1/800 (Deg-CALU) dilution in TBST. For the analysis of the antibody level temporal trends ELISA, the serum samples obtained from the control group were pooled together. Three replicas for each serum sample were tested. After a 1-hour incubation at room temperature, plates were washed and 50 µL/well of rabbit anti-IgY-peroxidase conjugate (Sigma-Aldrich, St. Louis, USA), diluted 1/30,000 in TBST, were added. After six washes with washing solution, 50 µL/well of substrate solution (Fast OPD, Sigma-Aldrich, St. Louis, USA) was added. Finally, the reaction was stopped after 20 mins with 25 µL/well of 2.5 mM H_2_SO_4_, and the and the optical density (OD) was measured at 450 nm using ELx808IU Ultra Microplate Reader (Bio-Tek Instruments, UK). 

Western blot analysis was conducted as previously described [32]. Briefly, 10 µg of each recombinant antigen per lane were separated by electrophoresis on 12% Bis-Tris Novex gels in NuPAGE® MES SDS Running Buffer (GE Healthcare, UK). Proteins were transferred to nitrocellulose membrane using a Xcell II blot module (GE Healthcare, UK), following the manufacturer´s instructions. Individual lanes of the membrane were excised and blocked by incubation in 5% w/v dried skimmed milk in PBST at 4 °C for 12 h and washed in PBST afterwards. Sera from each vaccination group of day 28 post-vaccination were used as primary antibodies, sera from the same vaccination group but of day 0 were used as negative controls. Sera were diluted 1/100 in PBS and incubated for two hours at RT. The membrane was washed and incubated in rabbit anti-IgY-peroxidase conjugate (Sigma-Aldrich, St. Louis, USA), diluted 1/30,000 in PBS for 1 h at RT, followed by washing and colorimetric development with SIGMAFAST™ 3.3´-DAB (Sigma-Aldrich, St. Louis, USA).

### 2.10. Statistical Analysis

Antibody levels between vaccinated and control groups were compared by Mann-Whitney U test (*p* < 0.05) using SPSS (IBM^®^ SPSS^®^ Statistics® v23). Nymphs and dead or missing mites were removed from the analysis for the effects on reproduction. 

A generalized linear mixed model (GLMM) was performed to determine the group differences based on a Negative Binomial distribution and log as link function. The model included the total number of eggs laid per individual mite (oviposition) as the dependent variable. The treatment group was included as a fixed effect and hen number and assay replicate as random factors. For this analysis SPSS (IBM^®^ SPSS^®^ Statistics^®^ v23) software was used.

### 2.11. Ethics Approval

The experiment using hens was conducted under the regulations of a UK Home Office Project Licence; the experimental design was ratified by the Experiments and Ethics Committee of the Moredun Research Institute (MRI), UK.

## 3. Results and Discussion 

Vaccine development is a promising tool for the control of *D. gallinae*. However, limited knowledge is available about its proteome. Omics tools have provided new databases to allow the identification of PRM proteins and probable antigens [20,26]. To our knowledge, this is the first work that characterize the proteomes of fed and unfed *D. gallinae* adults and nymphs.

### 3.1. Proteomics Analysis of D. gallinae Development Stages and Feeding Status

**Figure 1:**
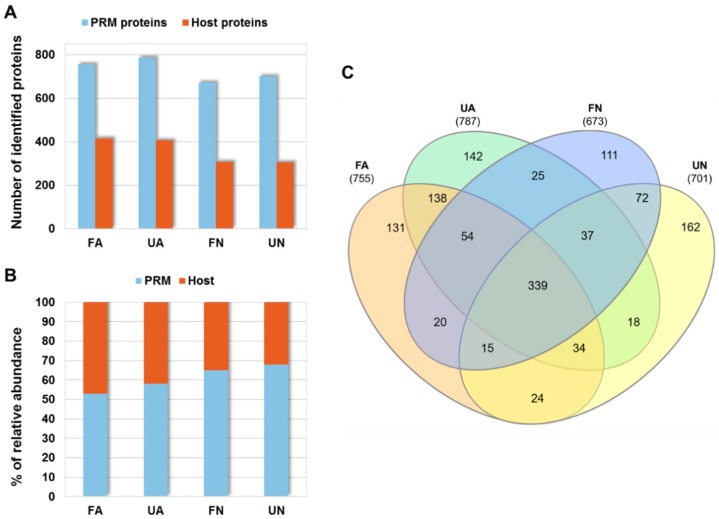
Proteomics results in PRM. (**A**) Proteins were identified with FDR < 0.01 and at least two PSMs per protein in at least one of the analyzed samples. (**B**) Percentage of relative abundance for PRM and host proteins calculated with respect to the total number of PSMs detected in each sample group. (**C**) Venn diagram showing PRM protein distribution between different groups analyzed. FA: engorged female adult mites, UA: non-fed female adult mites, FN: engorged proto- and deutonymphs, UN: unfed proto- and deutonymphs.

After proteomics analysis of PRM samples, as described in Materials and Methods, a total of 1322 PRM and 627 host proteins were identified, respectively (Supplementary Table S1). The number of identified proteins varied between experimental groups (fed adults (FA), unfed adults (UA), fed nymphs (FN) and unfed nymphs (UN)) ranging from 673 to 787 and from 304 to 414 for PRM and host proteins, respectively (Figure 1A). As expected, the proportion of host proteins present in mites increased with feeding and aging (Figure 1B). The same PRM proteins were shared in several experimental groups with 339 proteins found in all groups and 131, 142, 111 and 162 proteins that were identified exclusively in FA, UA, FN and UN, respectively (Figure 1C). 

**Figure 2:**
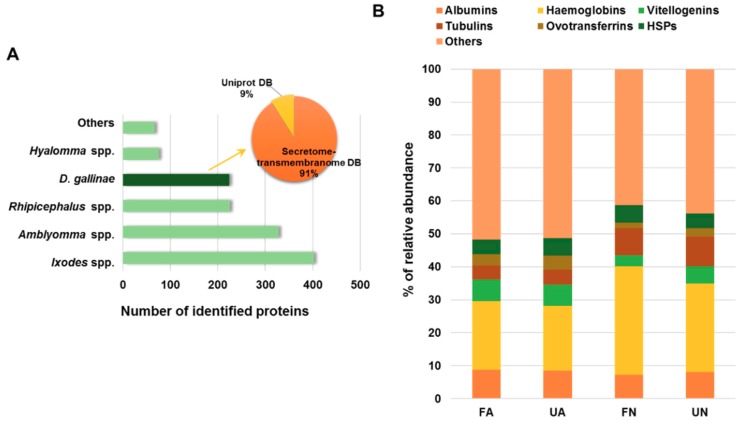
Distribution of PRM and host proteins identified. (**A**) Database assignation distribution of PRM proteins. (**B**) Percent distribution of host proteins calculated with respect to the total number of host PSMs detected in each sample group. FA: engorged female adult mites, UA: non-fed female adult mites, FN: engorged proto- and deutonymphs, UN: unfed proto- and deutonymphs.

From the PRM protein identified, 17% were proteins assigned to *D. gallinae*, whereas the rest of proteins were mainly assigned to *Ixodes*, *Amblyomma* and *Rhipicephalus* spp. (30%, 25% and 17%, respectively) (Figure 2A). These results indicating a high degree of homology between *D. gallinae* and tick proteins and a good quality of the data obtained because only the 6% of protein sequences present in the database constructed for searching belonged to *D. gallinae* (10,602 entries from a total of 181,866 entries). Moreover, data confirm the validity of the predicted secretome and transmembranome of *D. gallinae* [26] since the 91% of the *D. gallinae* assigned proteins match to entries from this database (204 of 224 *D. gallinae* proteins) (Figure 2A). Regarding host proteins, haemoglobins were the most abundant proteins identified, representing 21%, 19%, 33% and 26% of the total of host PMSs detected in FA, UA, FN and UN, respectively (Figure 2B). Host heat shock proteins were identified constituting around 5% of the total host proteins identified indicating the hens stress response as result of the mite bite (Figure 2B).

### 3.2. Functional Analysis of Identified Proteins in D. gallinae Development Stages and Feeding Status

**Figure 3:**
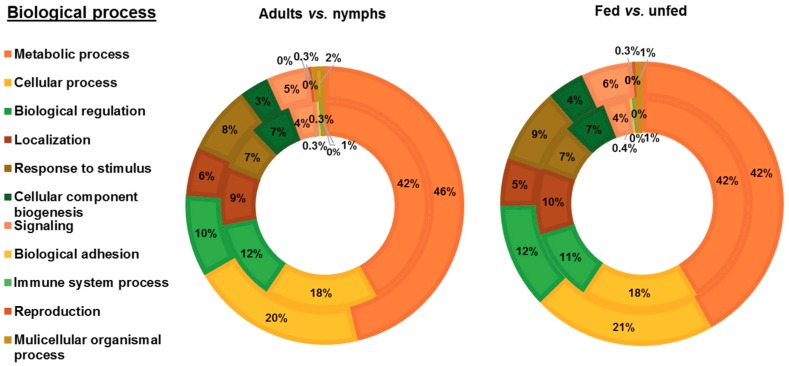
Biological process categorization of PRM proteins identified after proteomics analysis. Proteins were functionally annotated and grouped according to biological process using Blast2GO software and manual search. The same protein could have several GO annotations, a fact that was included in the results shown in the graphs. Left picture: Distribution of proteins identified in female adult PRM (inner circle) and nymph PRM (outer circle). Right picture: Distribution of proteins identified in fed PRM (inner circle) and unfed PRM (outer circle).

**Figure 4:**
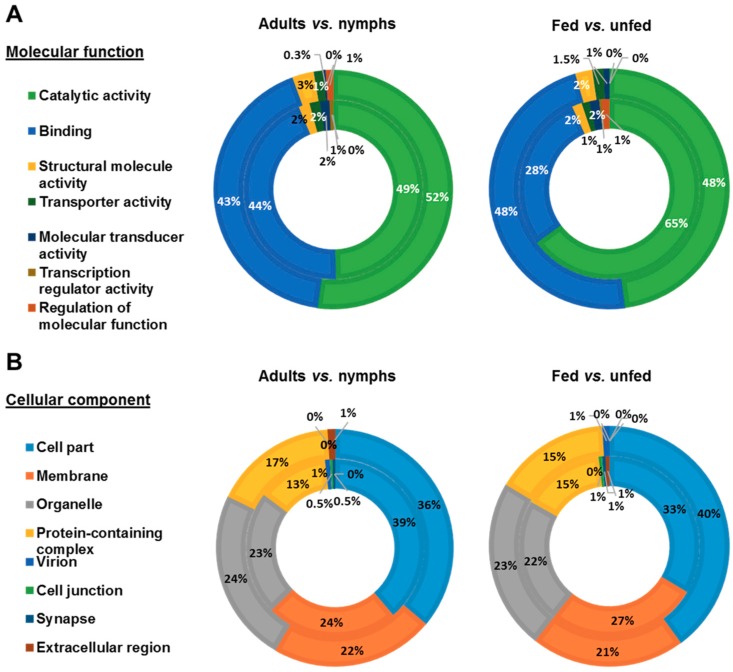
Molecular function and cellular component categorization of PRM proteins identified after proteomics analysis. Proteins were functionally annotated and grouped according to molecular function and cellular component using Blast2GO software and manual search. The same protein could have several GO annotations, a fact that was included in the results shown in the graphs. (**A**) Molecular function categorization. Left picture: Distribution of proteins identified in female adult PRM (inner circle) and nymph PRM (outer circle). Right picture: Distribution of proteins identified in fed PRM (inner circle) and unfed PRM (outer circle). (**B**) Cellular component categorization. Left picture: Distribution of proteins identified in female adult PRM (inner circle) and nymph PRM (outer circle). Right picture: Distribution of proteins identified in fed PRM (inner circle) and unfed PRM (outer circle).

PRM proteins identified were functionally annotated using Blast2GO software and manual search. Blast2GO search revealed 392 successful protein annotations and manual GO search offered 594 additional annotations. To compare between development stages, proteins identified in fed and unfed female adults, and in fed and unfed nymphs, were grouped in adults and nymphs groups, respectively. To compare the feeding status of mites, proteins identified in fed female adults and fed nymphs, and in unfed female adults and unfed nymphs were grouped in fed and unfed groups, respectively. GO annotations for proteins corresponding to each female adults, nymphs, fed and unfed groups were distributed according to Biological Process (BP) (Figure 3), Molecular Function (MF) (Figure 4A) and Cellular Component (CC) (Figure 4B). Most of the identified PRM proteins were involved in metabolic and cellular processes and biological regulation (Figure 3) participating in molecular functions related to binding or catalytic activity (Figure 4A). Regarding the cellular component, cell part, membrane, organelle and protein-containing complex were the most abundant localizations (Figure 4B).

Although protein ontology distribution in the different groups was similar, several differences can be observed. Biological processes involved in localization and cellular component biogenesis were over-represented in adults mites with respect to nymphs and in fed with respect to unfed mites, whereas the response to stimulus and signalling were found under-represented (Figure 3). Catalytic activity was the molecular function most relevant in mites reaching its maximum representation in fed mites (65%) where the value of binding was the lowest of the four groups (28%) (Figure 4A). Around a quarter of the proteins identified were part of the membrane with greater representation in adults and fed mites, compared to nymphs and unfed mites (Figure 4B). Proteins corresponding to extracellular region were undetectable in adults and fed mites (Figure 4B). 

**Figure 5:**
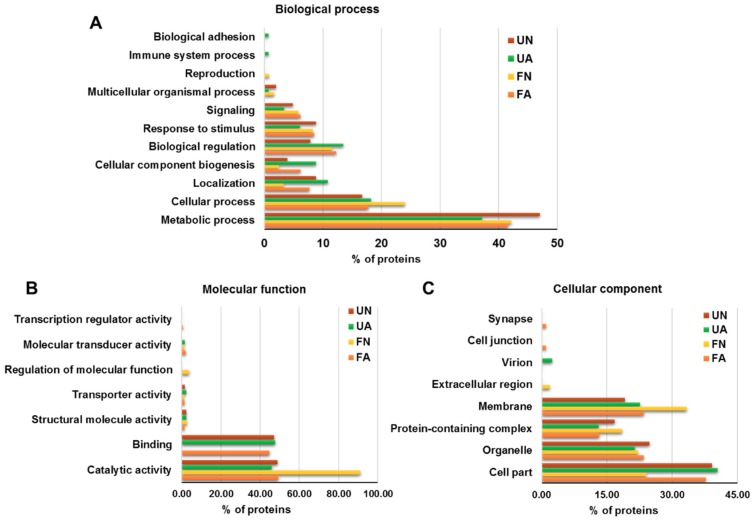
GO analysis of PRM proteins detected exclusively in each group analyzed. Proteomics analysis and DB search resulted in the identification of 131, 142, 111 and 162 proteins exclusively in FA, UA, FN and UN, respectively. These proteins were functionally annotated and grouped according to BP (**A**), MF (**B**) and CC (C) using Blast2GO software and manual search. The same protein could have several GO annotations, a fact that was included in the results shown in the graphs. FA: engorged female adult mites, UA: non-fed female adult mites, FN: engorged proto- and deutonymphs, UN: unfed proto- and deutonymphs.

To appreciate functional differences between the four groups of mites under study, the ontology of the proteins identified exclusively in each of the groups was analyzed (Figure 5). Within the two most abundant biological processes, unfed nymphs showed the most represented metabolic process whereas that fed nymphs highlighted in cellular processes (Figure 5A). Almost all the proteins exclusively detected in FN showed catalytic activity (91%) and only this group showed regulation of molecular function (Figure 5B). Moreover, proteins of fed nymphs were those located mostly in membrane showing proteins corresponding to extracellular region (Figure 5C).

### 3.3. Characterization of PRM Candidate Protective Antigens

From 1322 PRM proteins identified, 26 showed statistical differences between at least two groups after χ^2^-test (*p* < 0.05) (Table 1), fifteen of them identified in PRM secretome – transmembranome DB [26]. Related with feeding status, peptidyl-prolyl cis-trans isomerase were significantly over-represented in fed adults with respect to unfed adults, as were vitellogenins 1 and 2, and chromatin remodelling complex whereas histamine release factor and ribosomal protein s7 were under-represented, in fed *vs.* unfed nymphs (Table 1). The rest of the statistical differences were related with the developmental status (Table 1).

Several of the differentially represented proteins identified in this work had been tested before as vaccine candidates: vitellogenin and cathepsins [18], tropomyosin and paramyosin [29] and histamine release factor [30]. These proteins have been evaluated as vaccine candidates using the in-vitro feeding device. From the rest of proteins, only calumenin isoform 2 was over-represented in adults *vs.* nymphs, both in fed and unfed status, (χ^2^, *p* = 0.047 (AF *vs.* NF), *p* = 0.035 (AU *vs.* NU)) and identified in the secretome – transmembranome DB. For this reason, Deg-CALU was selected as a candidate protective antigen together with Rhm-SUB as positive control due to its previously identified effects on mite mortality after feeding [22]. 

**Figure 6:**
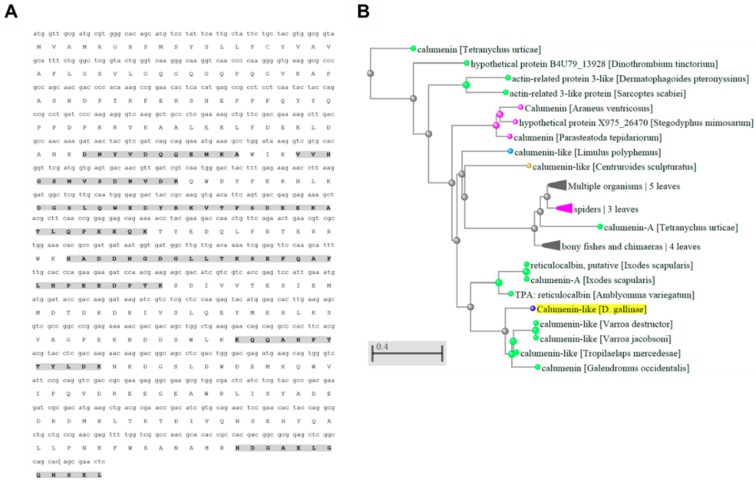
Deg-CALU protein coverage and phylogenetic analysis. (**A**) Calumenin isoform 2 coding sequence (isotig18930) [26]. Translated amino acid is shown with its corresponding nucleotide triplet. Peptides identified by RP-LC-MS/MS are marked (grey shadow). (**B**) Phylogenetic tree for Deg-CALU was constructed using the Blastp tool from BLAST (https://blast.ncbi.nlm.nih.gov/Blast.cgi).

Deg-CALU protein coverage based in peptide identification from proteomics data was 34.8% (Figure 6A). Phylogenetic analysis revealed that Deg-CALU is taxonomically related to calumenin-like proteins from other Mesostigmata mite species (Figure 6B). Calumenin is located in membranes, amongst other subcellular compartments and participates in metabolic process and binding. This ontology is in concordance with the most abundant biological process and molecular function identified in PRM proteins (Figure 3 and Figure 4).

### 3.4. Immune Response to Vaccination

**Figure 7:**
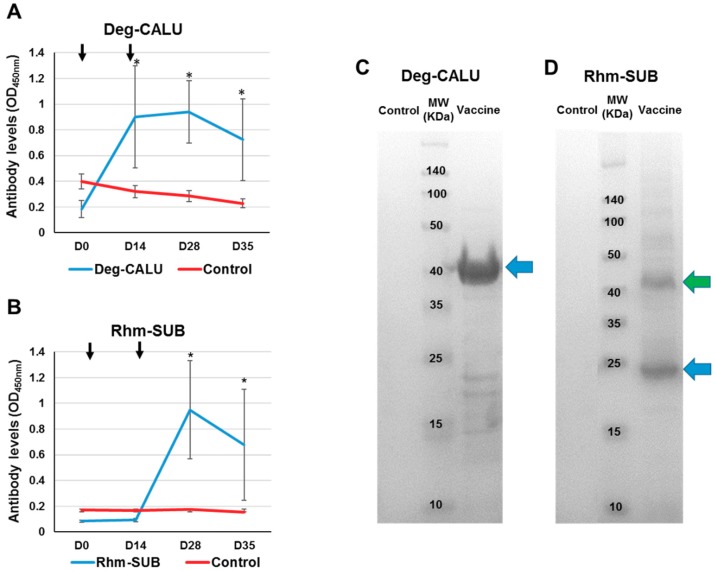
Characterization of the immune response against recombinant vaccines. Antibody levels were determined by ELISA in vaccinated and control hens against Deg-CALU (**A**) and Rhm-SUB (**B**). Serum samples were collected before each vaccination (arrows) (D0 and D14), during mite infestation (D28) and at the end of the experiment. Antibody titers are represented as the average OD450nm ± SD of three replicates and compared between vaccinated and control groups by Mann-Whitney U test (* *p* < 0.05). Serum samples for control group are pooled. Characterisation by Western Blot of anti-Deg-CALU IgY (**C**) and anti-Rhm-SUB (**D**). Ten µg of protein were loaded per well in a SDS-12% polyacrylamide gel. The gel was used for Western Blot analysis (WB) using sera of vaccinated and control hens collected on day 28 after V1. The position of the recombinant proteins is indicated with blue arrows. The green arrow indicates the potential presence of Subolesin dimers.

**Figure 8:**
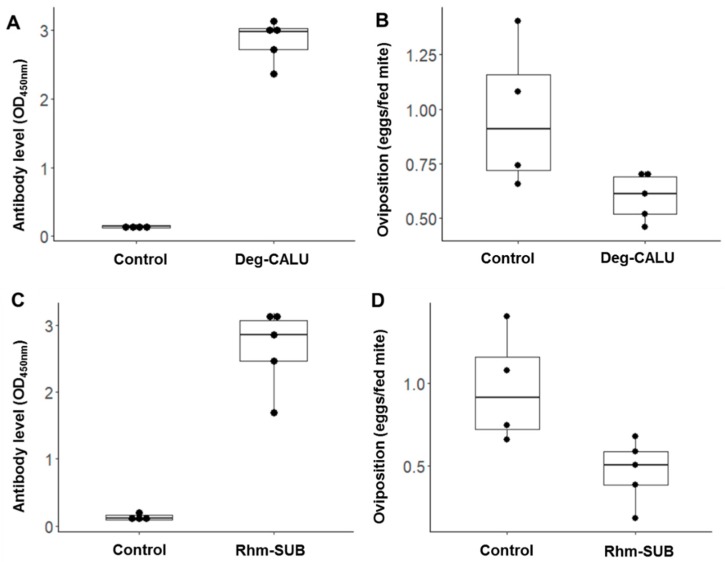
Comparison of antibody level and oviposition amongst groups. Antibody levels at the moment of infestation were determined by ELISA in vaccinated and control hens against Deg-CALU (**A**) and Rhm-SUB (**C**). Antibody titers are determined as the average OD450nm of three replicates and compared between vaccinated and control groups by Mann-Whitney U test (* *p* < 0.05). Oviposition of the Deg-CALU group (**B**) and Rhm-SUB (**D**) are shown to be compared with the control group. Oviposition is calculated as eggs laid/number fed adult viable female mites counted at day 7 of monitorization. Effect in oviposition is statistically significant in both groups (GLMM, *p* < 0.01).

Vaccination with Deg-CALU and Rhm-SUB triggered an antigen-specific IgY immune response in hens. Despite individual variation in the immune response for vaccinated hens was observed, the immune response of vaccinated hens was significantly higher than the control group (Mann-Whitney U test, *p* < 0.05). The serum IgY antibody response against Deg-CALU increased after immunization and remained higher than controls until the last bleeding (35 days after first immunisation) (Figure 7A and Figure 8A). The first administration of Rhm-SUB vaccine showed no increase in the antibody level compared to the control group; it was necessary to administer a second dose on day 14 to stimulate the immune response, reaching its peak on day 28 (Figure 7B and Figure 8C). This immune response to injected Subolesin is different from that observed by Harrington et al., 2009 [22], where two peaks in the antibody levels were observed. The difference in immune response to Subolesin between the current study and previous work might be attributable to one of these three differences in study design: the source of the Subolesin, the amount of injected antigen (20 μg first dose/40 μg second dose vs. 50 μg single dose), or the adjuvant used (Montanide^®^ ISA 71 VG, a poultry adjuvant, vs. Montanide^®^ ISA 50 V2, a livestock adjuvant). The serum recognition of Deg-CALU (Figure 7C) (37.7 kDa) and Rhm-SUB (Figure 7D) (18.7 kDa) by antigen-specific IgY present in the serum was also demonstrated by Western Blot. Serum anti-Rhm-SUB IgY also indicated the presence of Rhm-SUB multimers and some potential protein degradation products, a common effect with Akirin and Subolesin [22,37]. 

### 3.5. Vaccines Protective Effect

Vaccines against ectoparasites are not designed to prevent infestations but to reduce arthropod populations by affecting their feeding, reproduction and development after feeding on immunized animals and ingesting specific antibodies which interact with the target protein function [38].

Vaccination affected the mite reproduction in two ways: reducing the proportion of fed mites laying eggs and reducing the number of eggs laid per fed mite (Table 2). The number of female fed mites which laid eggs was reduced by a 35% in the Deg-CALU group (mean = 17 ± 4%) and a 44% in the Rhm-SUB group (mean = 14.9 ± 5%) when compared to control group (mean = 26.7 ± 8%) (Table 2). In a pair-wise comparison between treatment groups and control group the difference in the means were statiscally significant when performed a Student´s T-test with equal variance for Rhm-SUB and the control group (*t* = −2.66, df = 8, *p* = 0.03); and a tendency was observed when the test was performed between Deg-CALU and control (*t* = −2.331, df = 7, *p* = 0.05). Mites fed on hens vaccinated with Deg-CALU showed average oviposition of 0.62 (±0.1) eggs per viable fed female (Figure 8B) mite while for those vaccinated with Rhm-SUB the average oviposition was 0.48 (±0.2) eggs per fed viable female mite (Figure 8D), giving a reduction of 38% and 52%, respectively, when compared to the control group (Table 2). The effect on the oviposition was statistically significant (GLMM, F = 7.518, *p* = 0.001) with the Deg-CALU group (F = −0.552, SE = 0.172, *p* = 0.001) and the Rhm-SUB group (F = −0.633, SE = 0.183, *p* = 0.001). Calumenin is a well-conserved secreted protein in mammals [39,40] which may be involved in homeostatic and pathologic processes by regulation of Ca2+ transportation and could participate in signal transduction [41]. In invertebrates, it has been found in the digestive tract of several parasitic nematodes [42,43]. Calumenin has been revealed to be necessary for fertility, locomotion and body size in *Caenorhabditis elegans* [42]. Vaccination with Subolesin and Akirin have also shown deleterious effects on the reproduction in multiple ectoparasite species [37]. This is the first study in which the reproductive effects of vaccination with calumenin are shown.

Vaccination did not affect mite feeding rates, mortality, egg hatchability and larval development. No significant effects on feeding rates and mite mortality have been observed with the Deg-CALU and Rhm-SUB vaccinations (Table 2). The feeding rates observed in this study are similar to the obtained rates observed in previous works that used the on-hen feeding device [34,35]. Subolesin has reduced the number of engorged female ticks fed on vaccinated cattle [44]. In a previous work [22], a mosquito ortholog of the tick Subolesin was used to vaccinate hens showing a 35.1% increased mite mortality during in-vitro feeding tests. However, in-vitro feeding tests have shown a high background mite mortality in previous studies [16]. The use of anticoagulants and the high temperatures needed for encouraging mite feeding poses variable background mortality in the in vitro test [35]. The on-hen feeding assay allows a more physiological feeding of the mites, as they directly fed from the skin of the bird, and feeding on untreated (no heparin) blood. 

## 4. Conclusions

The results obtained in this study provide the first description of the proteomes of different development stages and feeding status in the PRM. Based on this description, we selected a new vaccine candidate and evaluated its efficiency. The reduction in the oviposition observed in hens vaccinated with Deg-CALU and Rhm-SUB supports their consideration as protective antigens for the control of PRM.

## Figures and Tables

**Table 1:** Differentially Represented Proteins Associated with Feeding and Developmental Status.

Accession No. ^a^	Description	Fold Change ^b^	χ^2^ *p*-Value	Significance ^c^
isotig10396	Peptidyl-prolyl cis-trans isomerase	∞	0.040	FA *vs*. UA
		0	0.025	UA *vs*. UN
isotig11213	Vitellogenin 2	1.54	0.000	FN *vs*. UN
		1.44	0.001	UA *vs*. UN
isotig15430	Vitellogenin 2	1.63	0.014	FA *vs*. FN
		1.36	0.037	UA *vs*. UN
A0A0M4FBG8	Vitellogenin 1 (*D. gallinae*)	1.65	0.000	FN *vs*. UN
		0.70	0.006	FA *vs*. FN
S5GFP7	Vitellogenin 2 (*Neoseiulus cucumeris*)	0.38	0.019	FA *vs*. FN
		4.20	0.002	FN *vs*. UN
		3.00	0.027	UA *vs*. UN
B5B8U1	Histamine release factor (*D. gallinae*)	0.29	0.020	FN *vs*. UN
A0A131Y6P3	Ribosomal protein s7 (*Ixodes ricinus*)	0.27	0.031	FN *vs*. UN
		0.18	0.012	UA *vs*. UN
A0A023GGI9	Chromatin remodeling complex rsc subunit rsc1/polybromo (Amblyomma *triste*)	∞	0.044	FN *vs*. UN
A0A1R3S3F0	Paramyosin (*D. gallinae*)	0.45	0.018	FA *vs*. FN
Q2WBI0	Tropomyosin (*D. gallinae*)	0.35	0.026	FA *vs*. FN
isotig21684	Cathepsin l precursor	0.29	0.017	UA *vs*. UN
isotig16123	Cathepsin l	0.22	0.033	UA *vs*. UN
isotig20927	Cathepsin l	0	0.024	UA *vs*. UN
isotig21530	Cathepsin l-like	0	0.014	UA *vs*. UN
isotig21385	Cathepsin s-like	0	0.004	UA *vs*. UN
isotig18930	Calumenin isoform 2	3.33	0.047	FA *vs*. FN
		3.67	0.035	UA *vs*. UN
isotig12641	Protein npc2-like protein	0.27	0.031	UA *vs*. UN
isotig11090	Chymotrypsin b-like	0	0.049	FA *vs*. FN
		0	0.014	UA *vs*. UN
isotig21611	Cytotoxin-like protein	0	0.027	FA *vs*. FN
V5HC54	Beta-spectrin (*I. ricinus*)	3.33	0.047	FA *vs*. FN
A0A131XHW1	Beta-spectrin (*Hyalomma excavatum*)	3.33	0.047	FA *vs*. FN
isotig18820	Deoxyribonuclease II	∞	0.007	FA *vs*. FN
A0A131Y3B8	Uncharacterized protein (*I. ricinus*)	19.00	0.000	FA *vs*. FN
A0A1E1X6V0	Conserved plasma membrane protein (*A. aureolatum*)	3.25	0.025	FA *vs*. FN
		6.50	0.005	UA *vs*. UN
isotig08662	PREDICTED: uncharacterized protein LOC100908559	0.22	0.033	UA *vs*. UN
isotig13475	PREDICTED: uncharacterized protein LOC100900008	0	0.027	FA *vs*. FN

^a^ Uniprot accession number or accession number to the database created from the predicted secretome and transmembranome of *D. gallinae* [26]. ^b^ Calculated from PSMs data (Supplementary Table S1). ^c^ FA: engorged female adult mites, UA: non-fed female adult mites, FN: engorged proto- and deutonymphs, UN: unfed proto- and deutonymphs.

**Table 2:** Summary of Adult Female PRM Feeding Rates and Vaccine Efficacy.

Antigen	Hen	Fed Mites	Unfed Mites	Total	% Fed	Average Feeding ± SD	% Reduction	Laying Mites	Total Viable Mites	% Laying	Average Laying ± SD	% Reduction	Egg	Eggs/Fed Mite	Average Oviposition ± SD	% Reduction
Rhm-SUB	1	154	91	245	62.9	60 ± 7	15	20	135	15	15 ± 5	44 *	59	0.44	0.48 ± 0.2	52 *
	2	126	60	186	67.7			25	125	20			85	0.68		
	3	82	82	164	50.0			5	80	6			15	0.19		
	4	153	115	268	57.1			26	148	18			79	0.60		
	5	178	112	290	61.4			27	171	16			87	0.51		
Deg-CALU	6	230	84	314	73.2	65 ± 15	7	29	215	13	17 ± 4	35 *	106	0.49	0.62 ± 0.1	38 *
	7	253	84	337	75.1			40	240	17			155	0.65		
	8	194	78	272	71.3			29	186	16			99	0.53		
	9	66	108	174	37.9			15	65	23			47	0.72		
	10	135	62	197	68.5			23	128	18			93	0.73		
Control	16	202	45	247	81.8	70 ± 12		73	190	38	27 ± 8		281	1.48	1.01 ± 0.4	
	17	158	130	288	54.9			29	147	20			103	0.70		
	18	210	59	269	78.1			48	204	24			156	0.76		
	20	180	93	273	65.9			44	175	25			194	1.11		

Data shown are a compilation from the three assays. Feeding rates were scored on day 1 of the assay, immediately following removal of the feeding devices from the hens. Reproduction effects were scored on day 7. Data were analyzed statistically to compare results between mites fed on vaccinated and control hens by GLMM. Statistically significant results (*p* < 0.05) are marked (*). Fed mites = total of fully engorged adult female mites recovered. Unfed mites = total of unfed adult female mites counted after 3 h placed on the hen. % Fed reduction = −[1 − (Average Feeding vaccinated group/Average Feeding Control)] × 100. (Student t-test, * *p* ≤ 0.05). Laying mites = total of mites that had laid eggs at day 7. Total viable mites = total of mites which that did not die or escaped at day 7 of monitoring. % Reduction in laying mites = −[1 − (Average Laying mites vaccinated group/Average Laying mites control group)] × 100. Eggs = total accumulative count of eggs laid at day 7. Oviposition = eggs laid/number fed adult viable female mites. % Oviposition reduction = −[1−(Average Oviposition vaccinated group /Average Oviposition Control)] × 100. (GLMM, * *p* <0.05).

## Supplementary Materials

The following are available online at https://www.mdpi.com/2076-393X/7/4/190/s1, Table S1: Proteomics Results.
